# Econazole Exhibits In Vitro and In Vivo Efficacy Against *Leishmania amazonensis*

**DOI:** 10.3390/ph19010185

**Published:** 2026-01-21

**Authors:** Juliana Tonini Mesquita, Ingrid de Oliveira Dias, Andre Gustavo Tempone, Juliana Quero Reimão

**Affiliations:** 1 Pathophysiology Laboratory, Instituto Butantan, São Paulo 01246-902, SP, Brazil; jt_mesquita@hotmail.com; 2 Laboratory of Preclinical Assays and Research of Alternative Sources of Innovative Therapy for Toxoplasmosis and Other Sicknesses (PARASITTOS), Faculdade de Medicina de Jundiaí, Jundiaí 13202-550, SP, Brazil; ingrid.oliveira.dias@hotmail.com

**Keywords:** *Leishmania*, cutaneous leishmaniasis, econazole, drug repurposing, in vivo

## Abstract

**Background/Objectives:** Cutaneous leishmaniasis (CL) remains a major neglected tropical disease, and current chemotherapeutic options are limited by toxicity and emerging resistance. Repurposing azole antifungals is a promising approach, as they target ergosterol biosynthesis, a pathway also essential in *Leishmania* spp. This study investigated the antileishmanial potential of econazole through in vitro and in vivo assays. **Methods:** Econazole activity was evaluated against *Leishmania amazonensis* promastigotes and intracellular amastigotes using MTT and luminescence-based methods. Cytotoxicity in NCTC cells was determined to calculate the selectivity index (SI). Drug interactions with miltefosine were assessed by fixed-ratio isobologram analysis. In vivo efficacy was examined in BALB/c mice infected with *L. amazonensis* and orally treated with econazole (2.5, 5, or 10 mg/kg/day) for 28 days. Lesion development and parasite burden were monitored. Molecular docking simulations were performed using SwissDock. **Results:** Econazole showed potent in vitro activity, with EC_50_ values of 8.9 µM for promastigotes and 11 µM for intracellular amastigotes, and a CC_50_ of 31 µM. Isobologram analysis revealed additive interactions with miltefosine (ΣFIC 0.5–1.2; mean 0.95). In vivo, econazole reduced lesion size and parasite load, achieving up to 75% reduction at 10 mg/kg/day. Docking results suggested that econazole may inhibit sterol biosynthesis, potentially through interaction with 14α-demethylase. **Conclusions:** These findings provide the first evidence of econazole activity against *L. amazonensis* in vitro and in vivo. Its exploratory efficacy and compatibility with miltefosine support further investigation of econazole as a repurposed candidate for CL, including optimization of dosing strategies and combination regimens.

## 1. Introduction

Leishmaniasis is a group of neglected tropical diseases caused by protozoan parasites of the genus *Leishmania*, comprising more than 20 pathogenic species that infect humans. The disease presents a wide clinical spectrum, ranging from self-healing cutaneous lesions to mucocutaneous and potentially fatal visceral forms [1,2]. According to the World Health Organization, more than 1 million new cases of cutaneous leishmaniasis (CL) occur annually, predominantly in low- and middle-income countries [2]. CL is the most prevalent clinical form and represents a significant public health burden, particularly in countries in Africa and the Middle East [3]. The clinical outcome of CL is highly heterogeneous and depends on multiple factors, including the infecting *Leishmania* species, parasite burden, and host immune response [1,2,4,5].

Despite its global impact, the therapeutic arsenal available for the treatment of CL remains limited and suboptimal. First-line treatments rely mainly on pentavalent antimonials, amphotericin B, and miltefosine, all of which are associated with considerable limitations. These drugs often require prolonged systemic administration, are linked to severe adverse effects such as cardiotoxicity, nephrotoxicity, and hepatotoxicity, and frequently lead to poor patient adherence and treatment discontinuation [6,7,8]. Moreover, increasing reports of parasite resistance, particularly to antimonials, further compromise treatment efficacy [9]. Alternative strategies, including topical formulations, immunomodulatory approaches, and combination therapies, have been explored to reduce toxicity and improve outcomes; however, none has yet fully addressed the need for safe, effective, and accessible treatments suitable for use in primary healthcare settings [10]. This therapeutic gap is exacerbated by the limited interest of the pharmaceutical industry in developing new drugs for neglected tropical diseases, reinforcing the urgent need for innovative and cost-effective solutions [11].

In this context, drug repurposing has emerged as a promising strategy to accelerate the identification of new antileishmanial candidates by exploring new indications for existing, clinically approved drugs with well-characterized pharmacokinetic and safety profiles [12]. Among repurposed drug classes, azole antifungals have attracted considerable attention due to their well-established mechanism of action and broad clinical use [13]. Azoles inhibit sterol 14α-demethylase (CYP51), a key enzyme in the ergosterol biosynthesis pathway, which is essential not only for fungal cell membrane integrity but also for protozoan parasites such as *Leishmania* spp. and *Trypanosoma cruzi*. Inhibition of this pathway leads to altered membrane composition, increased permeability, and parasite death, making CYP51 an attractive therapeutic target for trypanosomatid infections [14].

Several azole derivatives, including ketoconazole and fluconazole, have been investigated for their antileishmanial potential, showing in vivo efficacy [15]. Econazole, an imidazole derivative widely used as a topical antifungal agent, represents an attractive but underexplored candidate for repurposing. In addition to its ability to inhibit ergosterol biosynthesis, econazole disrupts cell membrane integrity, increasing permeability and promoting leakage of intracellular contents [16]. Beyond its antifungal activity, econazole has shown bactericidal effects against *Mycobacterium tuberculosis*, including synergistic interactions with standard antitubercular drugs, highlighting its potential for repositioning in infectious diseases [17].

Importantly, our group previously demonstrated the in vitro antileishmanial activity of econazole against *Leishmania infantum*, revealing effects against promastigote and intracellular amastigote forms and identifying plasma membrane permeabilization as a key component of its mechanism of action [18]. However, despite these promising findings, the activity of econazole against *Leishmania amazonensis*, a major etiological agent of CL in the Americas and a species associated with severe and treatment-refractory lesions, has not yet been explored in vivo.

Based on this background, the present study aimed to evaluate the antileishmanial potential of econazole against *L. amazonensis* using a comprehensive experimental approach. We investigated its in vitro activity against promastigotes and intracellular amastigotes, assessed cytotoxicity and selectivity, examined its interaction with miltefosine, and evaluated its therapeutic efficacy in a murine model of CL. Additionally, molecular docking analyses were performed to provide mechanistic insights into the interactions of econazole with sterol 14α-demethylase. Together, these data aim to support the repurposing of econazole as a promising and accessible candidate for the treatment of CL.

## 2. Results

### 2.1. Anti-Leishmania Activity and Cytotoxicity

The in vitro activity of econazole against promastigote forms of *L. amazonensis* was assessed using the MTT colorimetric assay. After 48 h of incubation, 100% parasite death was observed at the highest concentration tested, and the EC_50_ value was lower than that of the reference drug miltefosine (Table 1). When tested against intracellular amastigotes, econazole displayed an EC_50_ value comparable to miltefosine (Table 1). The cytotoxicity of econazole toward NCTC cells was determined by MTT assay after 48 h of treatment, allowing calculation of the Selectivity Index (SI) based on the ratio between the CC_50_ in mammalian cells and the EC_50_ against amastigotes (Table 1).

Based on these results, combination assays and in vivo experiments were conducted with econazole.

### 2.2. Isobolographic Studies of Econazole and Miltefosine

**Figure 1:**
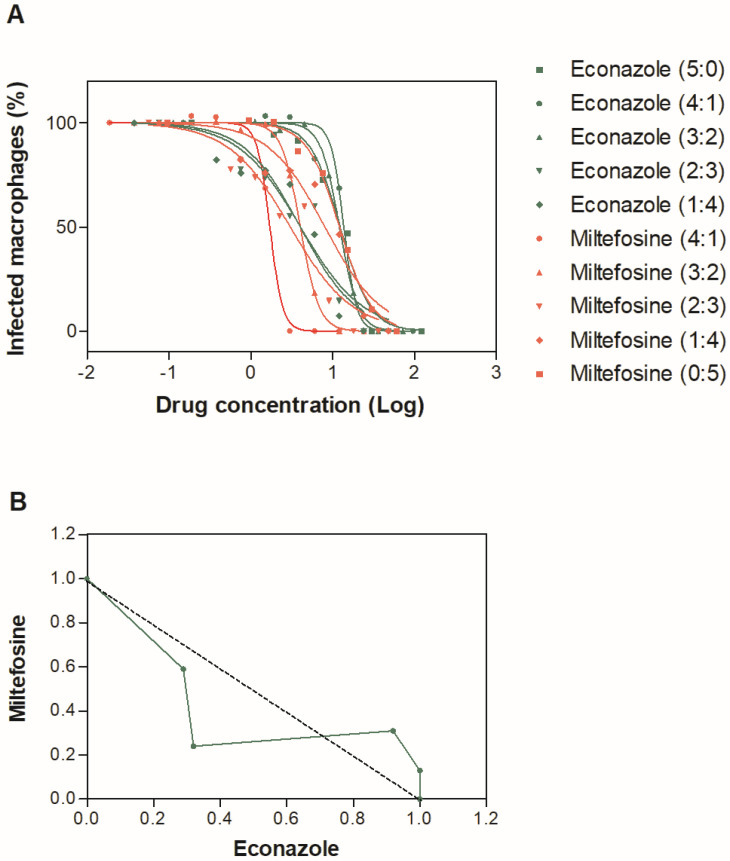
In vitro combinations of econazole and miltefosine against intracellular *L. amazonensis* amastigotes. (**A**) Dose–response curves for the combination of econazole and miltefosine against intracellular amastigotes of *L. amazonensis*. Representative experiment. (**B**) Isobologram based on ΣFIC values of econazole–miltefosine combinations against intracellular amastigotes of *L. amazonensis*. The dotted line represents the additive/indifferent interaction.

The combined effect of econazole and miltefosine was investigated against intracellular amastigotes using the modified fixed-ratio isobologram method after 48 h of incubation. Dose–response curves were generated for six fixed drug ratios (5:0, 4:1, 3:2, 2:3, 1:4, and 0:5), and representative curves are shown in Figure 1A. A gradual reduction in the EC_50_ values of each compound was observed as the proportion of the partner drug increased, indicating concentration-dependent modulation of activity.

ΣFIC values ranged from 0.56 to 1.23, with a mean ΣFIC (XΣFIC) of 0.95, classifying the interaction as additive/indifferent according to established criteria (0.5 ≤ ΣFIC ≤ 4). Although the 2:3 econazole/miltefosine ratio yielded a ΣFIC value close to the synergy threshold, no combination reached the cutoff for synergism (ΣFIC ≤ 0.5). These minor variations around the threshold likely reflect experimental and biological variability inherent to fixed-ratio combination assays rather than true synergistic interactions.

The isobologram analysis confirmed this interpretation, as all data points and the resulting trendline remained within the additive/indifferent interaction window (Figure 1B). Results represent the mean of two independent experiments, and no statistically significant synergistic or antagonistic interactions were detected under the experimental conditions tested.

### 2.3. In Vivo Efficacy of Econazole in L. amazonensis-Infected BALB/c Mice

**Figure 2:**
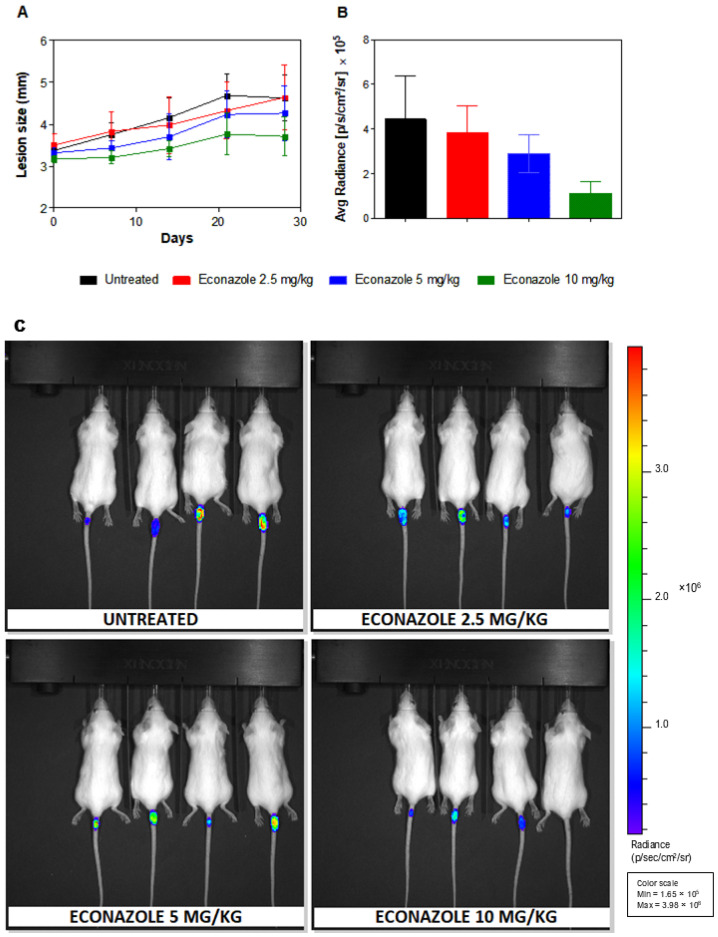
In vivo efficacy of econazole. Parasite burden and lesion size in *L. amazonensis*-infected BALB/c mice treated orally with econazole (2.5, 5, or 10 mg/kg/day) for 28 days (4 animals/group). (**A**) Lesion size (mean ± SD). Time zero indicates treatment initiation (five weeks post-infection). (**B**) Parasite burden assessed by lesion bioluminescence (mean ± SD). Units: photons/sec/cm^2^/sr. (**C**) Representative bioluminescence images of untreated and econazole-treated mice after 28 days of treatment.

The therapeutic efficacy of econazole was evaluated in BALB/c mice infected with *L. amazonensis*. Mice were inoculated at the tail base and, five weeks post-infection, allocated into four groups (4 animals/group): three groups received econazole orally at 2.5, 5, or 10 mg/kg/day for 28 consecutive days, and one group remained untreated (control). Lesion size and parasite burden were assessed. Lesion measurements revealed reductions of 4%, 11%, and 18% on day 14 of treatment for the 2.5, 5, and 10 mg/kg/day groups, respectively, compared with controls. On day 28, reductions reached 8% and 20% in the 5 and 10 mg/kg/day groups, respectively (Figure 2A). Parasite burden assessed by in vivo bioluminescence on day 28 (Figure 2B,C) showed reductions of 14%, 34%, and 75% in mice treated with 2.5, 5, and 10 mg/kg/day, respectively, compared with untreated animals. No statistically significant differences were observed among groups.

Throughout the 28-day treatment period, econazole was well tolerated at all tested doses. No clinical signs of toxicity, behavioral alterations, or treatment-related mortality were observed, including in animals receiving the highest dose (10 mg/kg/day). In addition, body weight remained stable and comparable to that of untreated control animals, indicating good tolerability of the oral treatment regimen.

### 2.4. Sequence Alignment of CYP51 from Leishmania Species

To support the use of sterol 14α-demethylase (CYP51) from *L. infantum* as a surrogate structure for molecular docking studies targeting *L. amazonensis*, a sequence alignment between the two orthologs was performed. The alignment revealed a high degree of conservation, with 97.29% amino acid sequence identity between CYP51 from *L. infantum* and *L. amazonensis* (Supplementary Figure S1). Importantly, residues associated with the catalytic core, substrate-binding cavity, and heme coordination were fully conserved. This high level of sequence identity supports the structural similarity between the two enzymes and justifies the use of the *L. infantum* CYP51 crystallographic structure as a reliable model for docking analyses involving *L. amazonensis*.

### 2.5. Structural Comparison and RMSD Analysis of CYP51 Enzymes

A structural comparison between CYP51 from *L. infantum* and its human homolog was performed to assess the degree of structural conservation and to support the comparative molecular docking analyses. Superposition of the two enzymes using the MatchMaker algorithm implemented in UCSF Chimera revealed a root-mean-square deviation (RMSD) of 1.10 Å calculated over 299 pruned atom pairs, indicating a high level of structural similarity within the conserved catalytic core of the enzyme.

When all aligned atoms were included in the calculation, the RMSD increased to 2.58 Å, reflecting expected conformational variability in flexible loop regions and terminal segments that are not directly involved in catalysis. Importantly, the spatial organization of the heme prosthetic group and the residues lining the substrate-binding cavity were largely conserved between the parasitic and human CYP51 enzymes (Supplementary Figure S2).

Despite this overall structural similarity, subtle differences were observed in regions surrounding the active site, particularly in peripheral residues that may influence ligand accommodation and stabilization. These local variations may contribute to differences in binding pose distribution and interaction patterns, potentially underlying the preferential accommodation of econazole within the parasitic CYP51 observed in the docking analysis. Collectively, these structural data support the use of *L. infantum* CYP51 as a suitable surrogate for *L. amazonensis* in structure-based studies and provide a structural framework for interpreting the qualitative docking results.

### 2.6. Docking Results

Molecular docking analysis indicated that econazole binds within the catalytic pocket of CYP51 in both the parasitic and human enzymes. In the parasitic system, econazole adopted binding poses localized near the heme group, a region critical for enzymatic activity.

A similar binding region was observed in the human CYP51; however, differences in the spatial arrangement of active-site residues resulted in distinct ligand orientations and interaction patterns. In the human enzyme, the predicted interactions appeared less optimally accommodated within the catalytic cavity, which may contribute to the lower predicted binding affinity compared to the parasitic enzyme.

**Figure 3:**
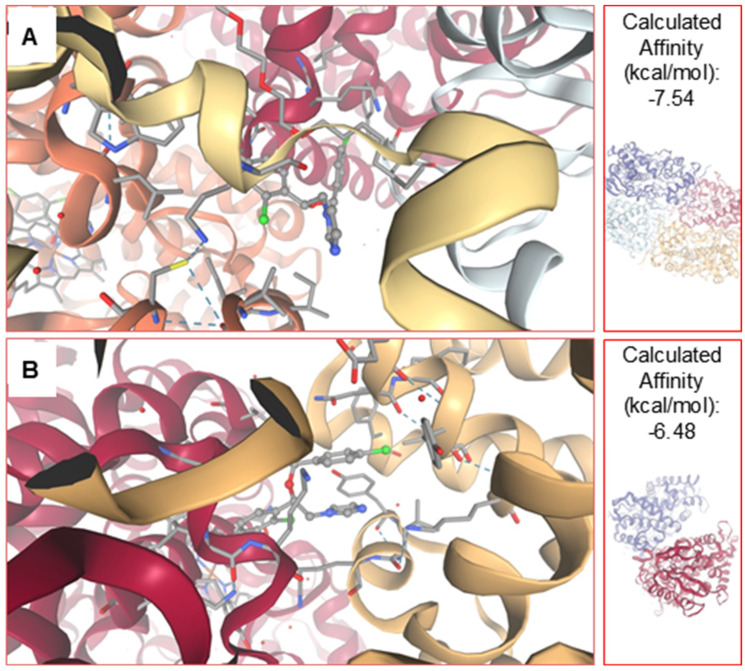
Comparative molecular docking analysis of econazole with sterol 14α-demethylase from *Leishmania* (**A**) and humans (**B**). Binding energy values (kcal/mol) were obtained from molecular docking simulations. More negative values indicate stronger ligand–target affinity.

The estimated binding free energy (ΔG) for econazole was −7.54 kcal/mol for the parasitic CYP51 and -6.48 kcal/mol for the human homolog. Although the difference in binding energy between the two enzymes was modest, the docking results revealed distinct pose distributions and interaction patterns within the active sites, suggesting a trend toward differential accommodation of econazole in the parasitic enzyme (Figure 3).

Analysis of docking pose convergence showed that the lowest-energy conformations clustered consistently within the catalytic pocket for both systems. Root-mean-square deviation (RMSD) values calculated among the top-ranked poses within the predominant cluster were below 2.0 Å, using the lowest-energy pose as reference, indicating good internal consistency and stability of the predicted binding modes.

Taken together, these findings support the plausibility of econazole interaction with CYP51 and provide qualitative mechanistic insight into its potential mode of action. However, the docking results are interpreted as exploratory and indicative of binding preference rather than definitive evidence of biological selectivity, underscoring the need for further validation through enzymatic assays and molecular dynamics simulations.

## 3. Discussion

Econazole belongs to the azole class of chemotherapeutic agents, which are widely used for the treatment of fungal infections due to their broad-spectrum activity against yeasts and filamentous fungi [16]. Beyond their antifungal use, azole derivatives have been increasingly investigated for repurposing against protozoan infections, including leishmaniasis [19,20]. Previous work demonstrated the anti-leishmanial activity of imidazole derivatives against *L. infantum*, identifying econazole as the most active compound against intracellular amastigotes when compared to the reference drug miltefosine [18]. The present study extends these findings to *L. amazonensis*, reinforcing the potential of econazole as a repurposable antileishmanial agent.

The ergosterol biosynthetic pathway represents a validated therapeutic target in fungi and trypanosomatid parasites, including *Leishmania* and *Trypanosoma cruzi* [14]. Azoles inhibit sterol 14α-demethylase (CYP51), a key enzyme in this pathway, leading to sterol depletion, membrane destabilization, and parasite death [19,21]. In addition to CYP51 inhibition, previous experimental evidence suggests that econazole may induce membrane damage and oxidative stress in promastigotes, contributing to its antiparasitic activity [18]. These multifactorial effects support the relevance of investigating econazole within the broader context of ergosterol-targeting agents.

Molecular docking analyses were employed as a complementary, hypothesis-generating approach to gain mechanistic insight into the interaction between econazole and parasitic CYP51. The docking results revealed a trend toward stronger predicted binding of econazole to the parasitic enzyme compared with the human homolog, although the difference in binding free energy was modest. The predicted binding mode was consistent with canonical azole–CYP51 interactions, including accommodation of the ligand within the hydrophobic catalytic pocket and proximity of the imidazole nitrogen to the heme iron, a hallmark of azole-mediated inhibition [22]. Structural differences between parasitic and human CYP51, particularly in residues lining the active site, may influence ligand stabilization and contribute to the observed preference. Importantly, these findings are interpreted as qualitative indicators of binding trends rather than definitive evidence of biological selectivity, and they primarily serve to contextualize econazole within the well-established class of azole CYP51 inhibitors.

The predicted interaction of econazole with the human CYP51 observed in the docking analysis is consistent with previous experimental evidence. Azole antifungals are known to interact with CYP51, primarily through coordination of the imidazole nitrogen to the heme iron [23]. Biochemical studies have shown that azoles can inhibit human CYP51 in vitro, although typically with lower potency than their fungal or protozoan counterparts, contributing to their therapeutic selectivity [14]. Moreover, econazole has been reported to inhibit multiple human CYP isoforms in vitro, supporting its ability to bind mammalian CYP enzymes at the molecular level [24]. In this context, the docking results should be interpreted as reflecting a plausible binding interaction rather than definitive evidence of functional inhibition or clinical relevance. This reinforces the need to consider selectivity, exposure, and safety margins when evaluating econazole as a repurposed antileishmanial candidate.

In vitro experiments demonstrated that econazole exhibits activity against both promastigote and intracellular amastigote forms of *L. amazonensis*, corroborating and extending previous observations in *L. infantum*. Among the azole derivatives previously evaluated, econazole consistently displayed activity against the clinically relevant intracellular stage, whereas compounds such as clotrimazole and bifonazole lacked efficacy against amastigotes [18]. Although econazole showed a lower selectivity index compared with miltefosine, its antiparasitic efficacy against intracellular parasites highlights a relevant profile within the azole class.

The relatively low selectivity index of econazole (SI = 2.8), particularly when compared with miltefosine (SI = 13.4), warrants careful consideration. While a low SI may raise concerns regarding safety, in vitro selectivity does not necessarily translate directly into in vivo toxicity. Notably, econazole was well tolerated in infected mice, with no observable signs of toxicity or body weight loss, even at the highest dose tested. Moreover, econazole is a clinically approved antifungal drug with an established safety record. These observations suggest that, although the SI highlights the need for further optimization, it does not preclude econazole from being considered a viable lead compound for antileishmanial drug repurposing, particularly in the context of optimized dosing strategies, alternative formulations, or combination therapy.

The in vitro combination studies between econazole and miltefosine revealed additive or indifferent interactions, with no evidence of antagonism. Although synergism was not observed, the absence of antagonistic effects indicates pharmacological compatibility between the two drugs. The additive interaction may be explained by their distinct but convergent effects on parasite membrane homeostasis: econazole primarily disrupts sterol biosynthesis through CYP51 inhibition, whereas miltefosine interferes with membrane lipid composition and cellular transport processes [25]. While both ultimately compromise membrane integrity, their actions occur at different molecular targets, allowing additive effects. Importantly, no in vivo combination study was performed, and these findings should therefore be interpreted as preliminary evidence of compatibility rather than proof of therapeutic benefit in vivo.

In vivo experiments provided exploratory evidence supporting the antileishmanial potential of econazole. Oral treatment of infected BALB/c mice for 28 days was associated with reductions in lesion size and parasite burden, with a trend toward greater effects at higher doses. However, due to the limited number of animals per group and the resulting variability, these differences did not reach statistical significance and should be interpreted with caution. Rather than demonstrating a definitive dose–response relationship, the observed pattern suggests a biological trend indicative of in vivo activity that warrants further investigation using larger sample sizes and optimized experimental designs.

Despite the observed in vivo effects following oral administration, it is important to acknowledge that econazole has limited oral bioavailability due to its physicochemical properties. Accordingly, the present results should not be interpreted as evidence of favorable systemic pharmacokinetics. Instead, the observed effects may reflect partial absorption, local tissue exposure, or preferential accumulation at the site of infection. Oral administration was employed as an exploratory and clinically relevant route to assess whether econazole could exert measurable antileishmanial effects in vivo. Given the cutaneous nature of the disease, alternative delivery strategies—such as topical formulations, lipid-based systems, or nanocarrier-mediated delivery—may be more appropriate to enhance local drug exposure while minimizing systemic toxicity.

Collectively, these findings demonstrate that econazole exhibits relevant in vitro and exploratory in vivo activity against *L. amazonensis* and shows pharmacological compatibility with miltefosine at the cellular level. While the current in vivo data are limited by sample size and experimental constraints, they provide a rationale for further preclinical investigation. Future studies should prioritize expanded in vivo evaluations with formulation optimization, and mechanistic validation through enzymatic and dynamic simulations. Within these limitations, the present study positions econazole as a promising candidate for repurposing and supports continued investigation of azole-based compounds in the context of CL.

## 4. Materials and Methods

### 4.1. Reagents and Compounds

Econazole nitrate was purchased from Sigma-Aldrich (St. Louis, MO, USA). Fetal bovine serum (FBS) was purchased from Gibco (Grand Island, NY, USA). One-Glo™ Luciferase Assay System and VivoGlo™ Luciferin were purchased from Promega (Madison, WI, USA). Miltefosine and other reagents, unless otherwise stated, were obtained from Sigma-Aldrich (St. Louis, MO, USA).

### 4.2. Parasites, Cells, and Animals

Wild-type *Leishmania amazonensis* promastigotes (MHOM/BR/1973/M2269) and luciferase-expressing mutants (LaLuci) were maintained in M-199 medium supplemented with 10% FBS and 0.25% hemin at 24 °C. LaLuci parasites were cultured with 32 µg/mL hygromycin B. Parasites were periodically passaged in female BALB/c mice by intradermal inoculation of 1 × 10^6^ promastigotes in the hind footpad or tail base, and amastigotes were recovered from lesion aspirates [26].

Bone marrow-derived macrophages (BMDMs) were obtained from female BALB/c mice by flushing femurs with R2030 medium (50% RPMI, 30% L929-conditioned medium, 20% FBS), followed by 7 days of culture at 37 °C with 5% CO_2_ [26]. Fibroblasts (NCTC clone 929, ATCC CCL1) were maintained in RPMI with 10% FBS under the same conditions and used for cytotoxicity assays. Female BALB/c mice (3–4 weeks old, 18–22 g) were obtained from the Instituto Adolfo Lutz animal facility. Animals were housed under standard conditions with food and water ad libitum. All procedures were approved by the Institutional Animal Care and Use Committee of Instituto Adolfo Lutz (CEUA-IAL 04-2016).

### 4.3. In Vitro Antileishmanial Activity

The half-maximal effective concentration (EC_50_) of econazole was determined against promastigotes and intracellular amastigotes. Promastigotes (1 × 10^6^/well, 96-well plates) were incubated with serial dilutions of compounds (starting concentration: 60 µM for econazole, 100 µM for miltefosine) for 48 h in M-199 supplemented with 10% FBS and 0.25% hemin. Viability was assessed by the MTT assay [26] at 570 nm using a FilterMax F5 Multi-Mode Microplate Reader.

For intracellular assays, BMDMs (1 × 10^5^/well) were seeded in 16-well plates (NUNC^®^) and infected with *L. amazonensis* promastigotes at a 20:1 ratio for 4 h, washed, and treated for 48 h with econazole or miltefosine [26]. At the end of the assay, the slides were stained with Giemsa and observed under an optical microscope. EC_50_ values were determined by counting 400 macrophages/well and evaluating the percentage of infected cells [27]. Untreated infected macrophages and DMSO-treated controls were included in all assays.

### 4.4. Cytotoxicity and Selectivity Index

Cytotoxicity was assessed in NCTC clone 929 fibroblasts (6 × 10^4^ cells/well, 96-well plates) exposed to compounds (initial concentration: 300 µM) for 48 h. Cell viability was determined by the MTT assay [26]. The selectivity index (SI) was calculated as SI = CC_50_ in mammalian cells/EC_50_ in intracellular amastigotes.

### 4.5. Drug Combination Studies

Drug interactions between econazole and miltefosine were evaluated in intracellular amastigotes using the fixed ratio isobologram method [28,29]. Drug dilutions were prepared in ratios of 5:0, 4:1, 3:2, 2:3, 1:4, and 0:5 relative to their individual EC_50_ values. The fractional inhibitory concentration (FIC) and the sum FIC (ΣFIC) were calculated. Interactions were classified as synergistic (ΣFIC ≤ 0.5), additive/indifferent (0.5 < ΣFIC ≤ 4), or antagonistic (ΣFIC > 4) [30].

### 4.6. In Vivo Efficacy in BALB/c Mice

Female BALB/c mice were infected intradermally at the tail base with 1 × 10^6^ LaLuci promastigotes in a final volume of 20 µL, as previously described [31]. Five weeks post-infection, when cutaneous lesions were fully established, animals were allocated to experimental groups (4 animals per group). Randomization was performed based on lesion size, ensuring that the mean lesion measurements were comparable across all groups at baseline. This procedure minimized potential allocation bias related to disease severity.

Mice were treated orally once daily with econazole at doses of 2.5, 5, or 10 mg/kg/day for 28 consecutive days. The selected dose range was based on previous in vivo studies demonstrating systemic efficacy and tolerability of econazole in murine infection models [17], as well as on pharmacological considerations aimed at evaluating a dose–response relationship while minimizing the risk of toxicity.

Econazole was suspended in a vehicle containing 15% Cremophor EL, 10% ethanol, and 75% PBS. Infected but untreated animals receiving vehicle alone were included as the control group.

Lesion size was measured weekly using a caliper (MARBERG) and expressed in millimeters (mm) [31]. Body weight was monitored throughout the treatment period as an indication of systemic toxicity. No animals were excluded from the study, and no deaths or dropouts occurred during the experimental period. All animals completed the treatment and were included in the final analysis.

At the end of the treatment regimen, parasite burden was quantified by in vivo bioluminescence imaging using an IVIS Spectrum system (Caliper Life Sciences, Hopkinton, MA, USA). Mice received an intraperitoneal injection of D-luciferin (75 mg/kg) and were anesthetized with isoflurane prior to imaging [31]. Photon emission was acquired and quantified as photons/s/cm^2^/sr using Living Image software (v4.3.1), as previously reported [31].

In accordance with the 3Rs principle (Replacement, Reduction, and Refinement), a standard antileishmanial drug was not included as a positive control in the present study, as this experimental model using miltefosine in BALB/c mice infected with *L. amazonensis* has been previously validated, employing the same infection protocol and outcome measures, including lesion evaluation and bioluminescence-based parasite burden assessment [32].

### 4.7. Molecular Docking

The three-dimensional structures of sterol 14α-demethylase (CYP51) were obtained from the Protein Data Bank (PDB). As no crystallographic structure of *L. amazonensis* CYP51 is currently available, the homologous enzyme from *L. infantum* (PDB ID: 3L4D) was used as a surrogate model. This choice was supported by sequence alignment and structural comparison analysis demonstrating a high degree of conservation between the two enzymes, particularly within the catalytic core and substrate-binding regions. The human CYP51 structure (PDB ID: 8SS0) was included for comparative purposes.

Structural superposition between parasitic and human CYP51 was performed using the MatchMaker algorithm implemented in UCSF Chimera. Root-mean-square deviation (RMSD) values were calculated to quantify structural similarity, both for pruned atom pairs representing the conserved core and for all aligned atoms, allowing assessment of global and local conformational differences [33].

Econazole (PubChem CID: 2065) was retrieved from the PubChem database and converted to a three-dimensional structure. Geometry optimization was performed using OpenBabel through force-field-based energy minimization to obtain a low-energy conformation prior to docking. Ligand protonation states were implicitly defined during preparation, consistent with standard docking workflows.

Protein structures were prepared in Chimera by removing crystallographic water molecules and co-crystallized ligands, followed by the addition of polar hydrogen atoms and assignment of Gasteiger partial charges. No additional structural optimization or molecular dynamics relaxation was applied to protein models.

Molecular docking simulations were conducted using SwissDock (https://www.swissdock.ch/), employing default parameters [34]. This approach was selected as a structure-based, exploratory method aimed at identifying plausible binding modes and relative affinity trends rather than generating quantitatively predictive binding free energies. Environmental parameters such as temperature, pH, ionic strength, solvent composition, and counterions were not explicitly modeled, as SwissDock performs docking in an implicit solvent framework.

Docking results were analyzed based on estimated binding free energy (ΔG), clustering of binding poses, and convergence of low-energy conformations within the catalytic pocket. For each system, the most populated and lowest-energy cluster was selected for further analysis. Root-mean-square deviation (RMSD) values among top-ranked poses within the dominant cluster were calculated using the lowest-energy pose as a reference to assess the consistency of predicted binding modes. Comparative analysis between the parasitic and human enzymes was performed to evaluate trends in binding preference and potential selectivity [35].

### 4.8. Statistical Analysis

EC_50_ and CC_50_ values were calculated by nonlinear regression of sigmoidal dose–response curves (GraphPad Prism 5.0). Differences between groups were analyzed by one-way ANOVA with Kruskal–Wallis. Statistical significance was defined as *p* < 0.05.

## 5. Conclusions

This study provides the first evidence of the in vitro and exploratory in vivo activity of econazole against *L. amazonensis*. Econazole demonstrated relevant activity against both promastigote and intracellular amastigote forms, confirming and extending previous observations reported for other *Leishmania* species. Although its selectivity index was lower than that of miltefosine, econazole demonstrated consistent antiparasitic effects against the clinically relevant intracellular stage.

In vivo oral treatment of infected BALB/c mice was associated with reductions in lesion size and parasite burden, with a trend toward greater effect at higher doses. However, due to the limited number of animals per group and the resulting variability, these findings should be interpreted as preliminary and indicative of biological activity rather than definitive evidence of dose-dependent efficacy.

Combination studies revealed additive interactions between econazole and miltefosine in vitro, with no evidence of antagonism, supporting pharmacological compatibility at the cellular level. Nonetheless, the absence of in vivo combination experiments precludes conclusions regarding therapeutic benefit in animal models, highlighting the need for further investigation.

Overall, these findings expand the potential of azole derivatives beyond their established antifungal use and support econazole as a promising lead for antileishmanial drug repurposing. Future studies should focus on optimizing formulation and delivery strategies, expanding in vivo evaluations with larger sample sizes, and integrating pharmacokinetic and mechanistic analyses. Such efforts will be essential to better determine the translational potential of econazole for the treatment of CL.

## Figures and Tables

**Table 1:** Evaluation of the EC_50_, CC_50_, and SI values of econazole and miltefosine against *Leishmania amazonensis* and mammalian cells.

Drug	*L. amazonensis* Promastigotes EC_50_ (µM) ± SD	*L. amazonensis* Amastigotes EC_50_ (µM) ± SD	Fibroblasts CC_50_ (µM) ± SD	SI
Econazole	8.9 ± 0.38	11.0 ± 0.1	31.2 ± 8.3	2.8
Miltefosine	12.5 ± 4.1	11.8 ± 3.2	157.8 ± 24.5	13.4

EC_50_: 50% effective concentration; CC_50_: 50% cytotoxic concentration; SD: standard deviation; SI: selectivity index (ratio CC_50_/EC_50_ for intracellular amastigotes). Cytotoxicity was assessed in NCTC clone 929 fibroblasts.

## Data Availability

The original contributions presented in this study are included in the article/Supplementary Material. Further inquiries can be directed to the corresponding authors.

## Supplementary Materials

The following supporting information can be downloaded at: https://www.mdpi.com/article/10.3390/ph19010185/s1. Figure S1: Multiple sequence alignment of sterol 14α-demethylase (CYP51) from *Leishmania infantum* (UniProt ID: A2TEF2) and *Leishmania amazonensis* (UniProt ID: Q2HWD2). The alignment was generated using Clustal Omega and visualized with MView. A high degree of sequence conservation was observed, with 97.29% amino acid identity between the two orthologs. Conserved residues within the catalytic domain, substrate-binding regions, and heme-coordinating motifs are highlighted, supporting the use of the *L. infantum* CYP51 structure as a surrogate model for molecular docking studies targeting *L. amazonensis*. Figure S2: Structural superposition of sterol 14α-demethylase (CYP51) from *Leishmania infantum* (beige) and *Homo sapiens* (cyan) generated using the MatchMaker algorithm in UCSF Chimera. The alignment yielded an RMSD of 1.10 Å across 299 pruned atom pairs, indicating a high degree of structural conservation within the catalytic core. The heme prosthetic group is shown in stick representation.
