# Supplementary material for: Econazole Exhibits In Vitro and In Vivo Efficacy Against Leishmania amazonensis
Source: Pharmaceuticals (Basel). 2026 Jan 21;19(1):185. doi: 10.3390/ph19010185 (PMC12845095; doi:10.3390/ph19010185)
Supplement: Supplementary file 1 [file pharmaceuticals-19-00185-s001.zip › pharmaceuticals-4077087-supplementary.pdf]

Supplementary Material

Econazole Exhibits *in vitro* and *in vivo* Efficacy Against *Leishmania amazonensis*

Juliana Tonini Mesquita [1], Ingrid de Oliveira Dias [2], Andre Gustavo Tempone [1,*] and Juliana Quero Reimão [2,*]

[1] Pathophysiology Laboratory, Instituto Butantan, São Paulo 01246-902 SP, Brazil
[2] Laboratory of Preclinical Assays and Research of Alternative Sources of Innovative Therapy for Toxoplasmosis and Other Sicknesses (PARASITTOS), Faculdade de Medicina de Jundiaí, Jundiaí 13202-550 SP, Brazil
* Correspondence: AT: andre.tempone@butantan.gov.br, JR: julianareimao@g.fmj.br

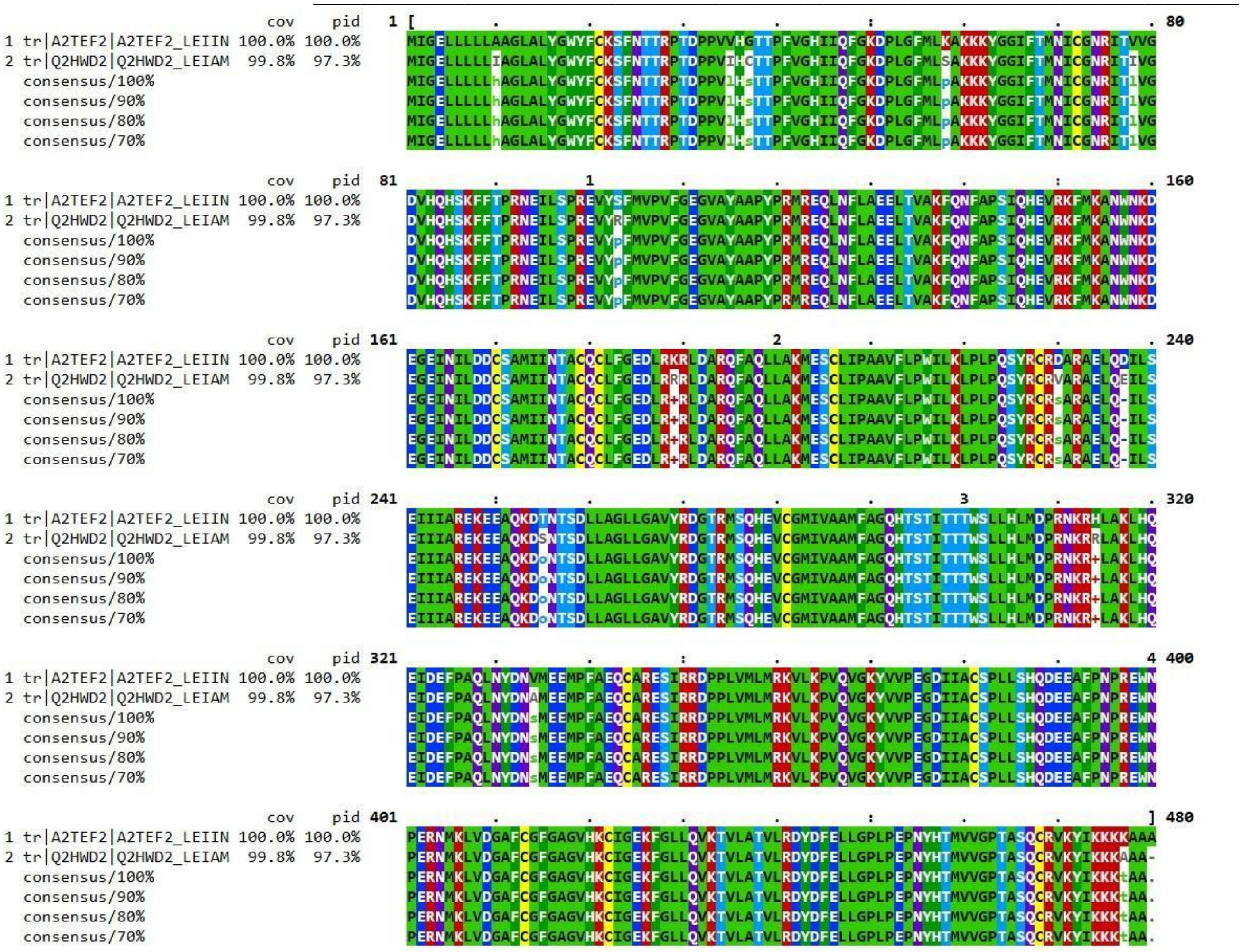

Figure S1: Multiple sequence alignment of sterol 14α-demethylase (CYP51) from *Leishmania infantum* (UniProt ID: A2TEF2) and *Leishmania amazonensis* (UniProt ID: Q2HWD2). The alignment was generated using Clustal Omega and visualized with MView. A high degree of sequence conservation was observed, with 97.29% amino acid identity between the two orthologs. Conserved residues within the catalytic domain, substrate-binding regions, and heme-coordinating motifs are highlighted, supporting the use of the *L. infantum* CYP51 structure as a surrogate model for molecular docking studies targeting *L. amazonensis*.

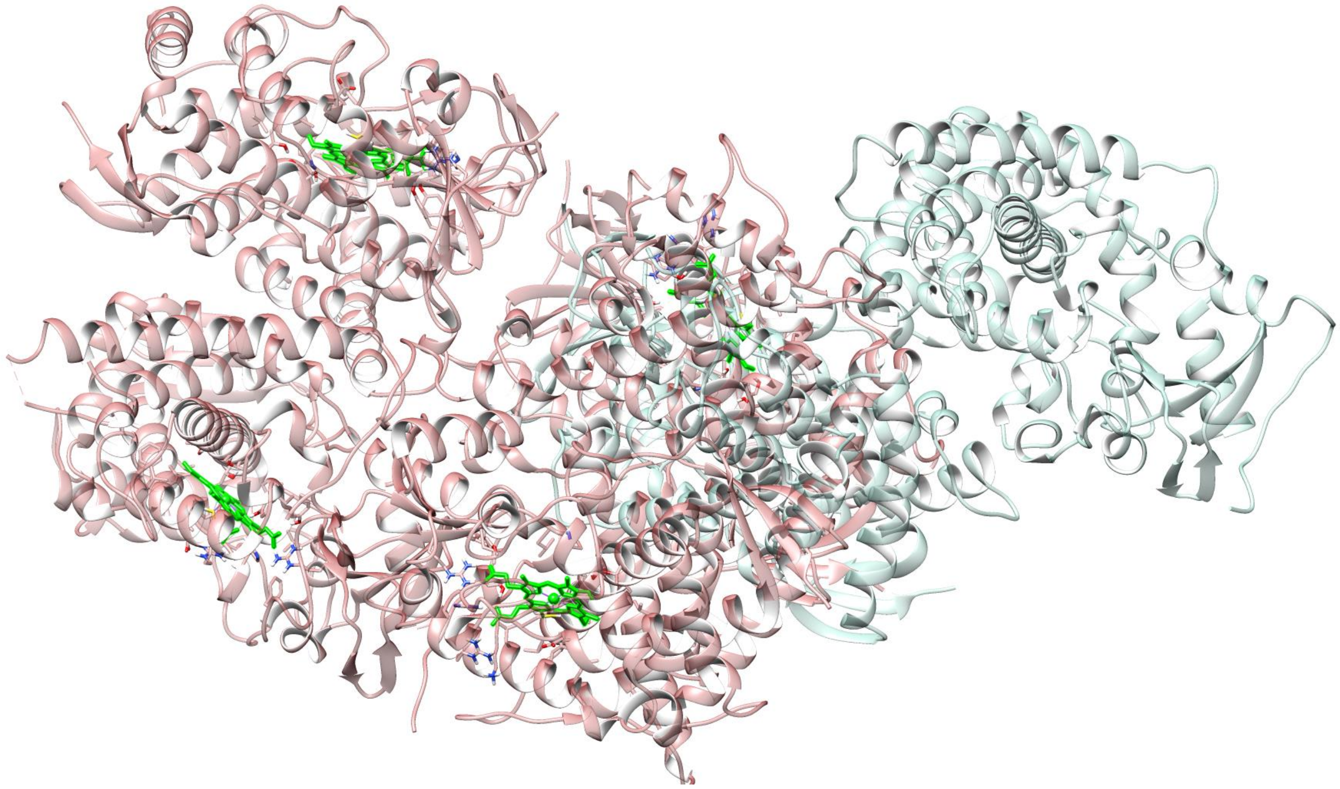

Figure S2: Structural superposition of sterol 14α-demethylase (CYP51) from *Leishmania infantum* (beige) and *Homo sapiens* (cyan) generated using the MatchMaker algorithm in UCSF Chimera. The alignment yielded an RMSD of 1.10 Å across 299 pruned atom pairs, indicating a high degree of structural conservation within the catalytic core. The heme prosthetic group is shown in stick representation.
